# Supplementary material for: The Intrinsic GDP/GTP Exchange Activities of Cdc42 and Rac1 Are Critical Determinants for Their Specific Effects on Mobilization of the Actin Filament System
Source: Cells. 2019 Jul 21;8(7):759. doi: 10.3390/cells8070759 (PMC6678527; doi:10.3390/cells8070759)

Supplementary Figure 1

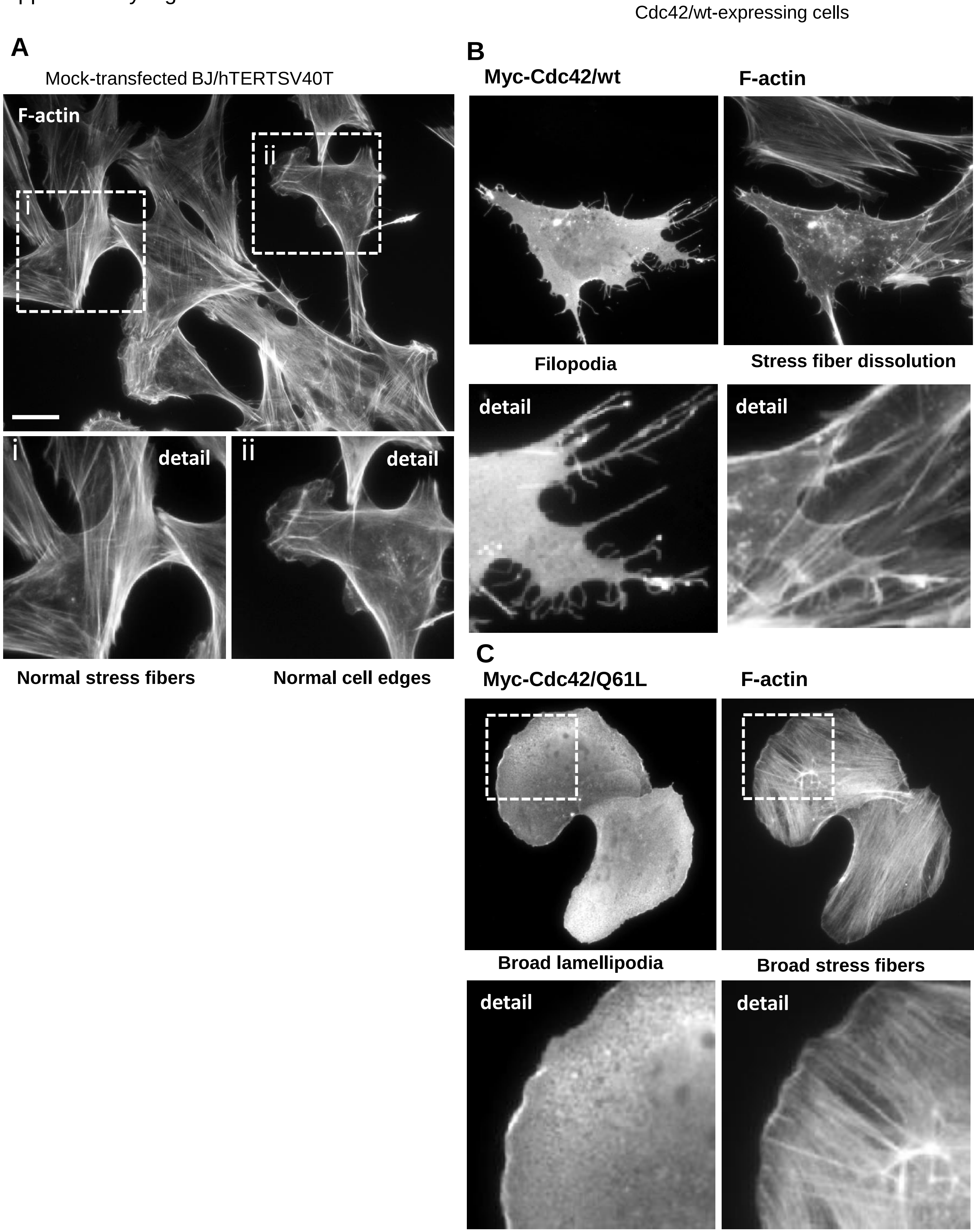

Supplementary Figure 2

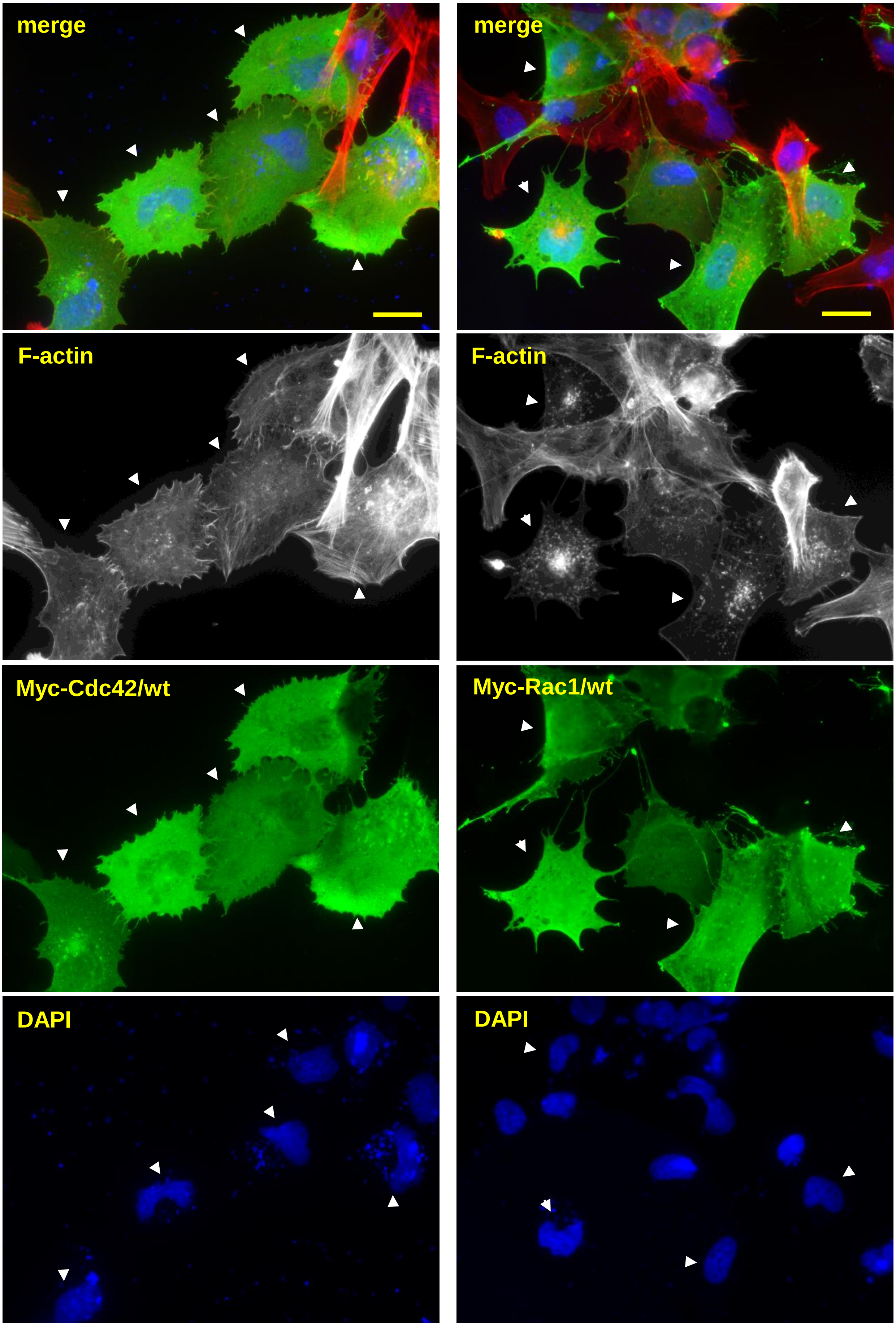

Supplementary Figure 3

A

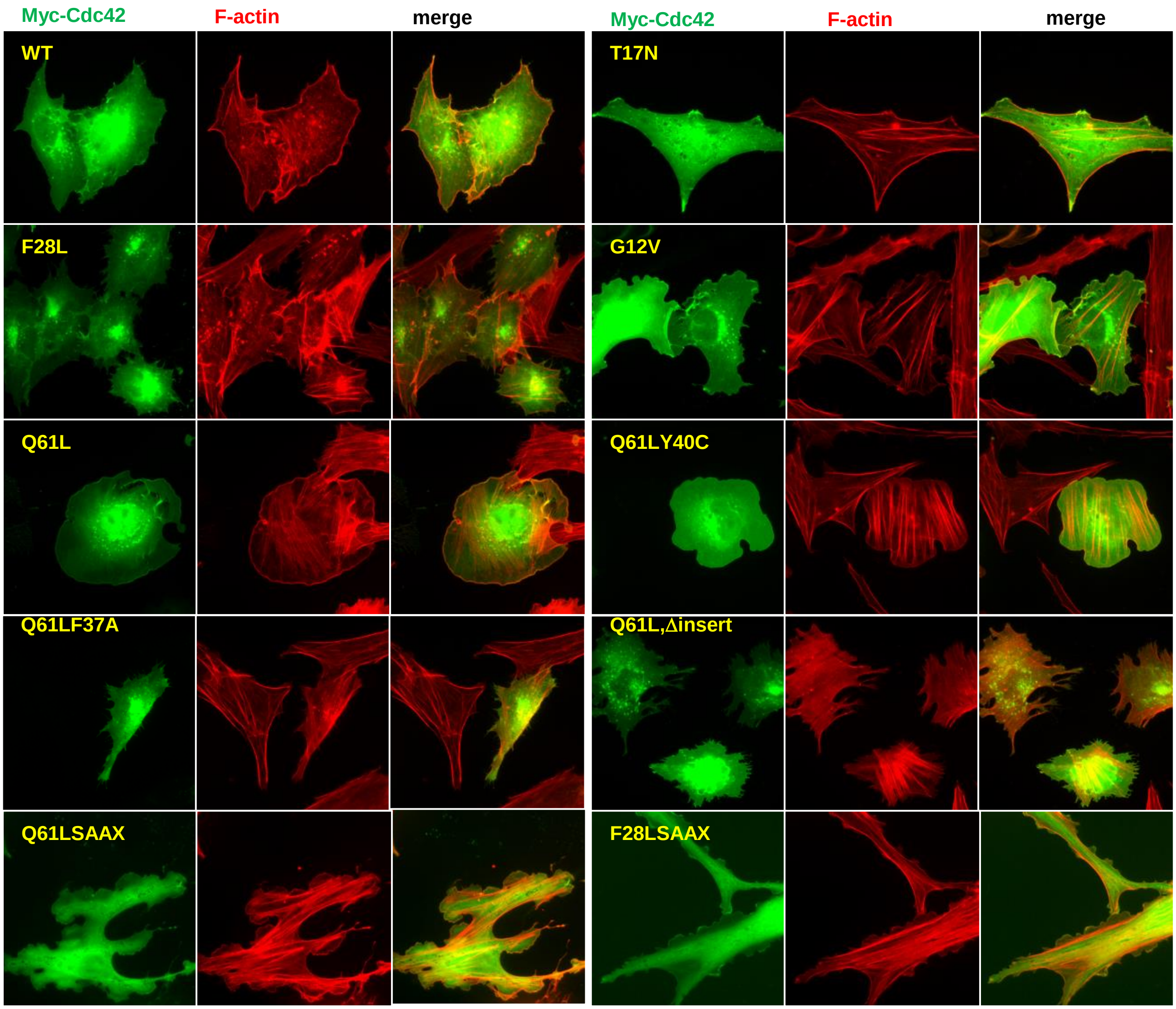

B

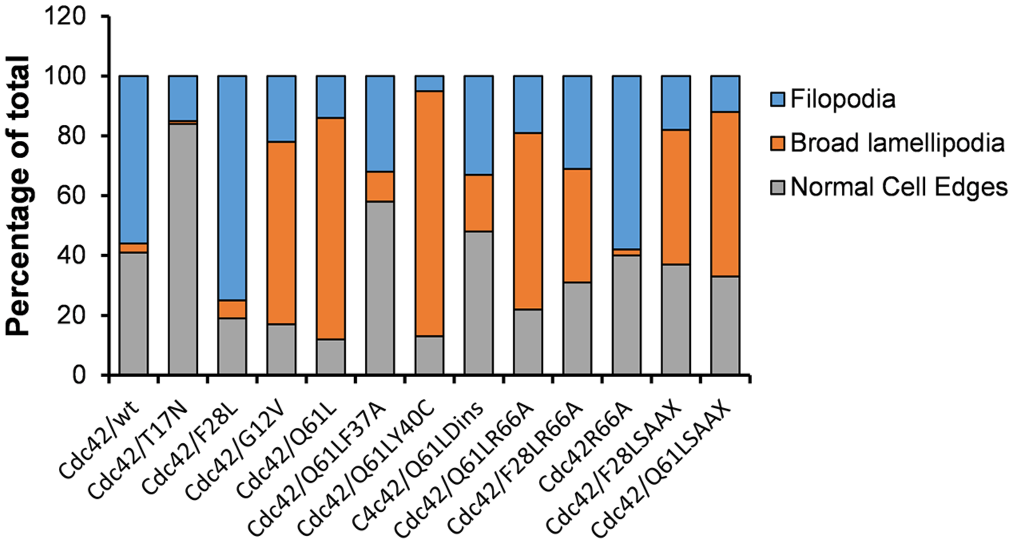

Supplementary Figure 4

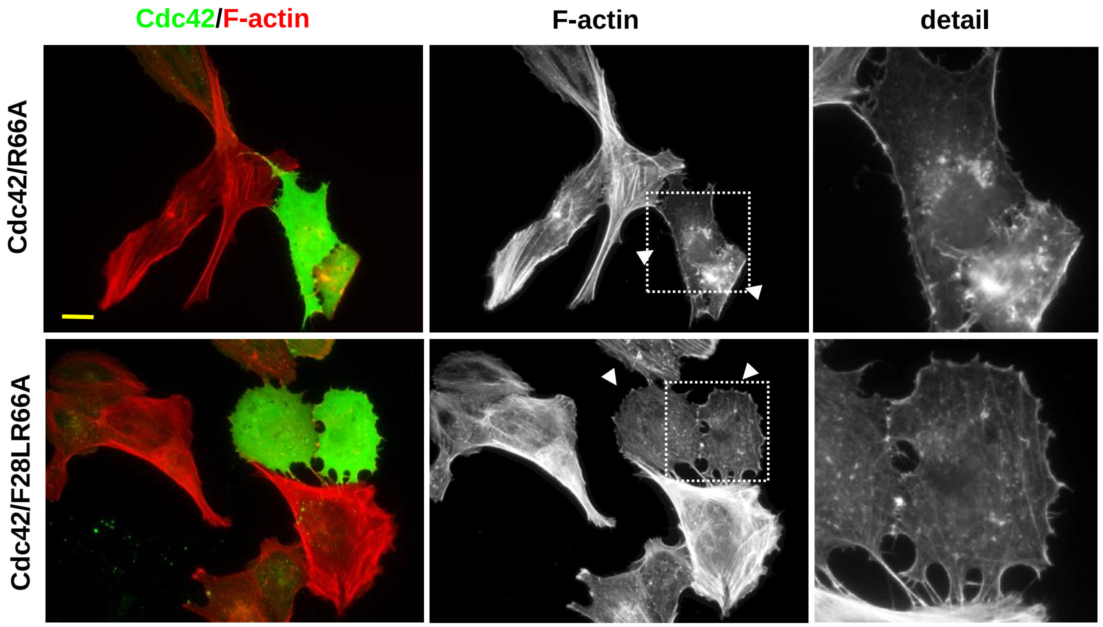

Supplementary Figure 5

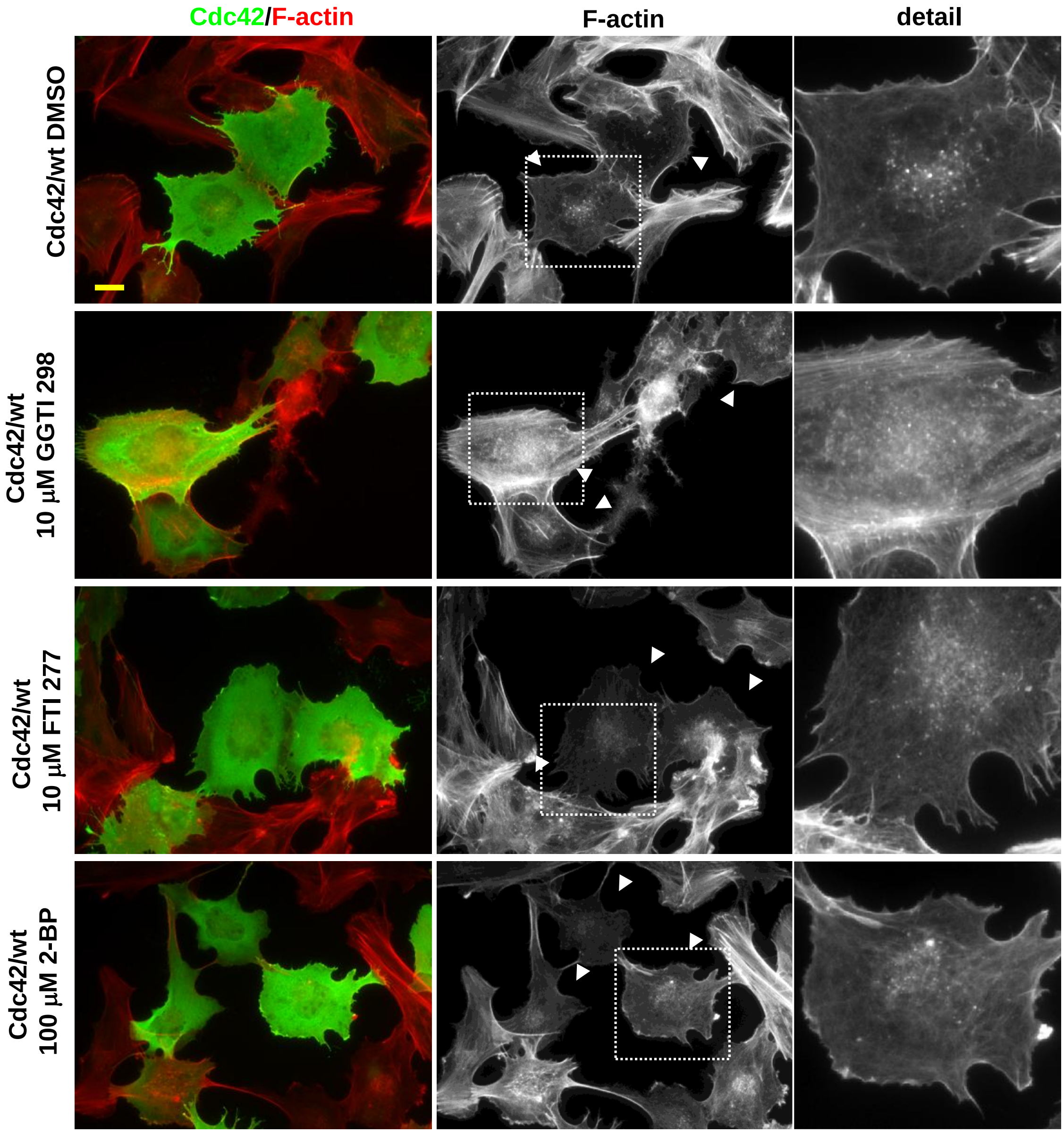

Supplementary Figure 6

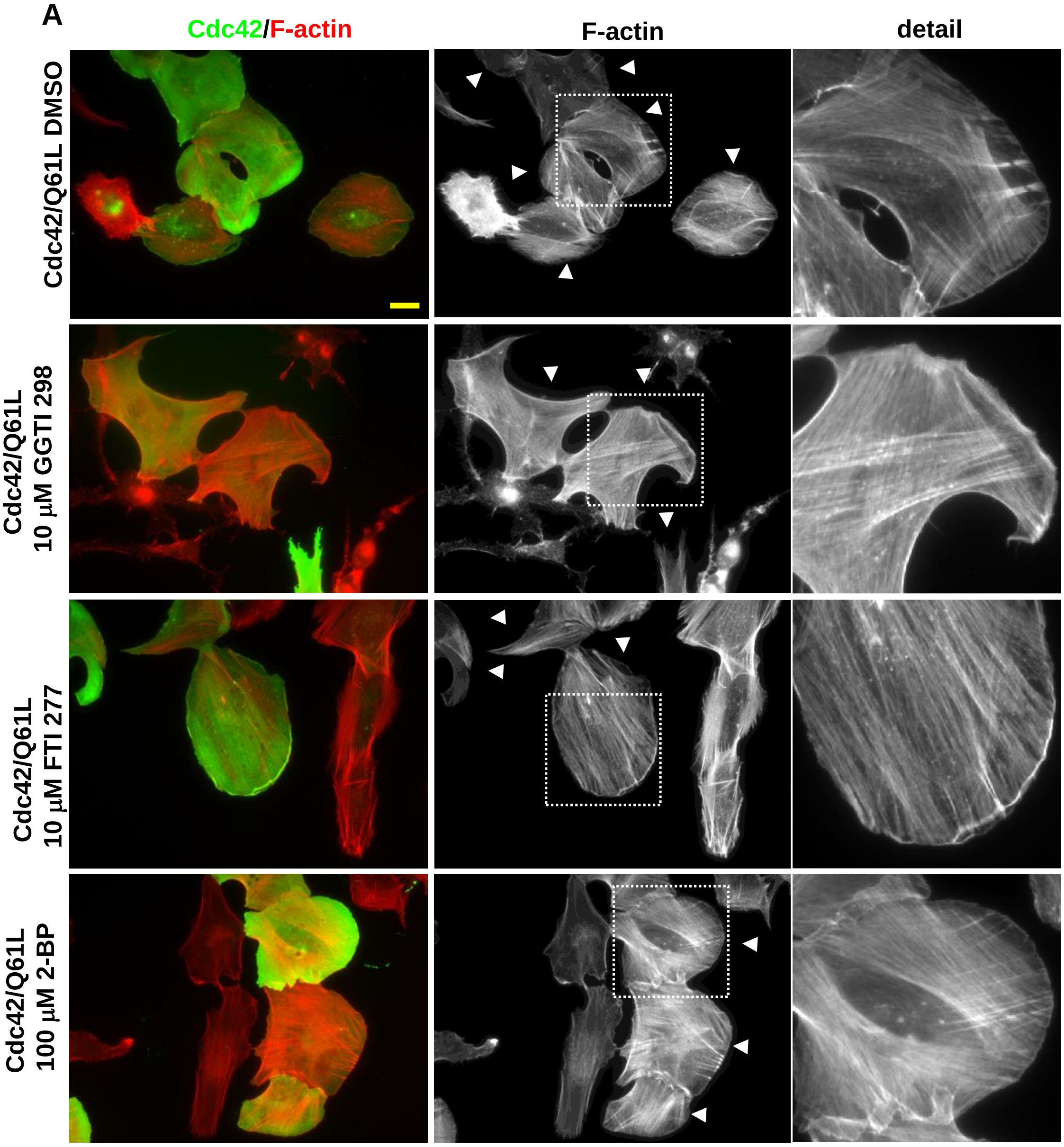

Supplementary Figure 7

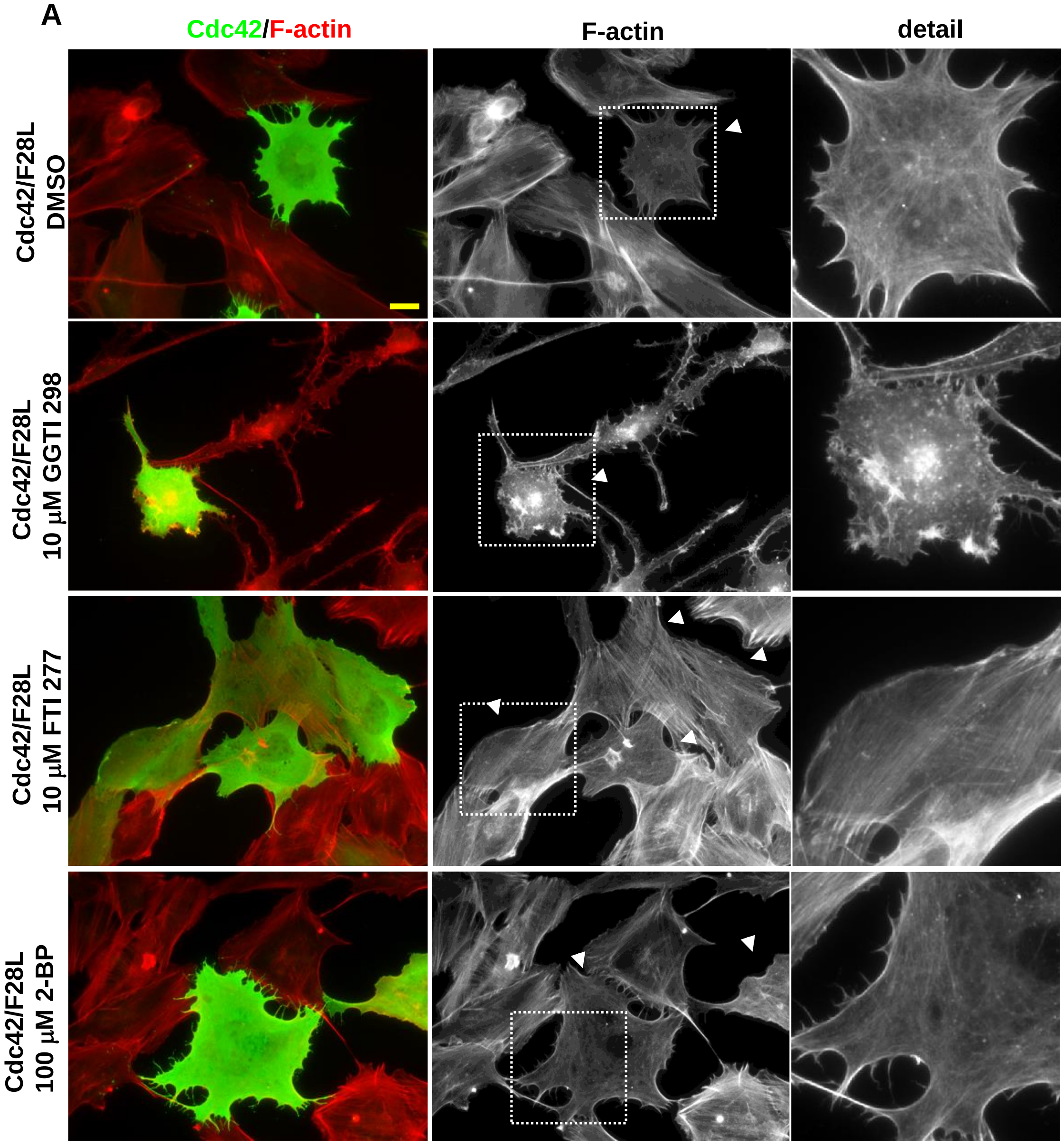

Supplementary Figure 8

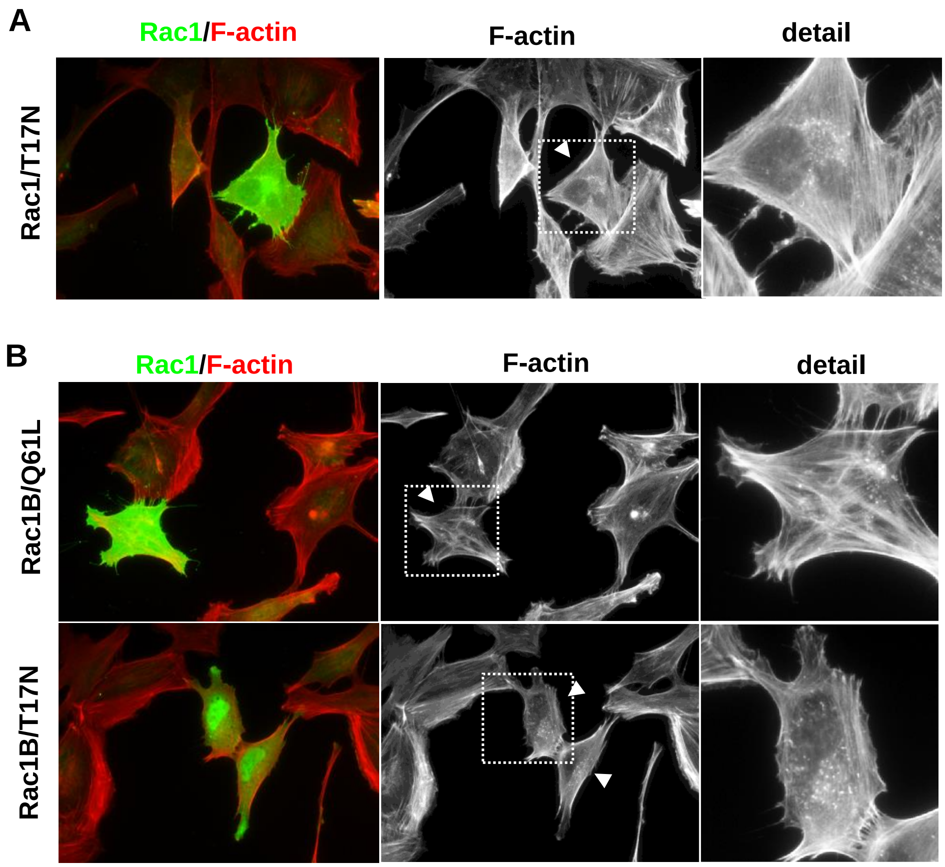

Supplementary Figure 9

A

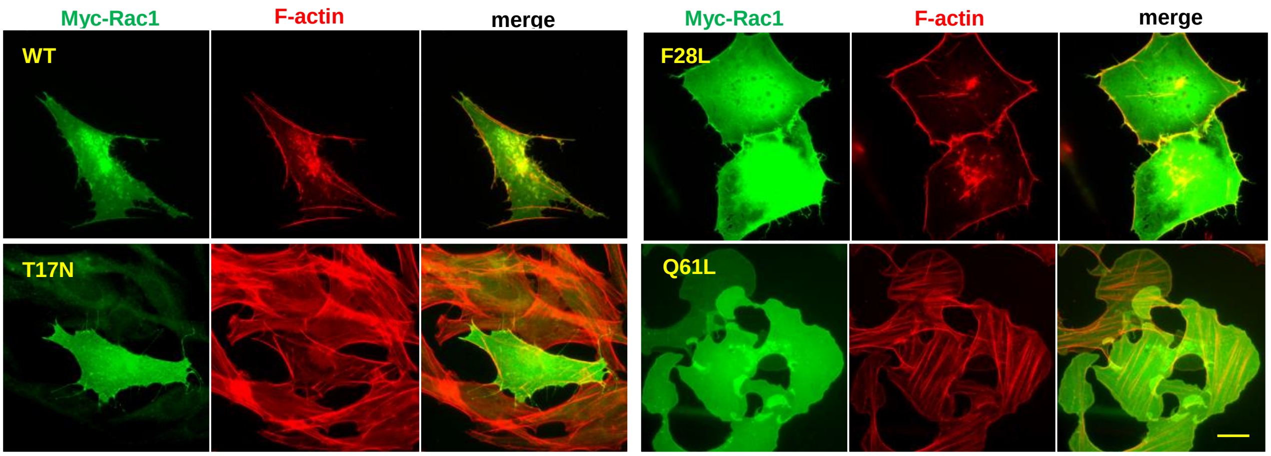

B

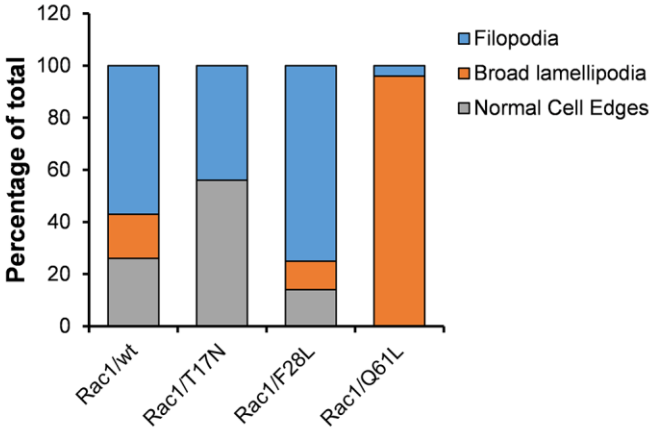

Supplementary Figure 10

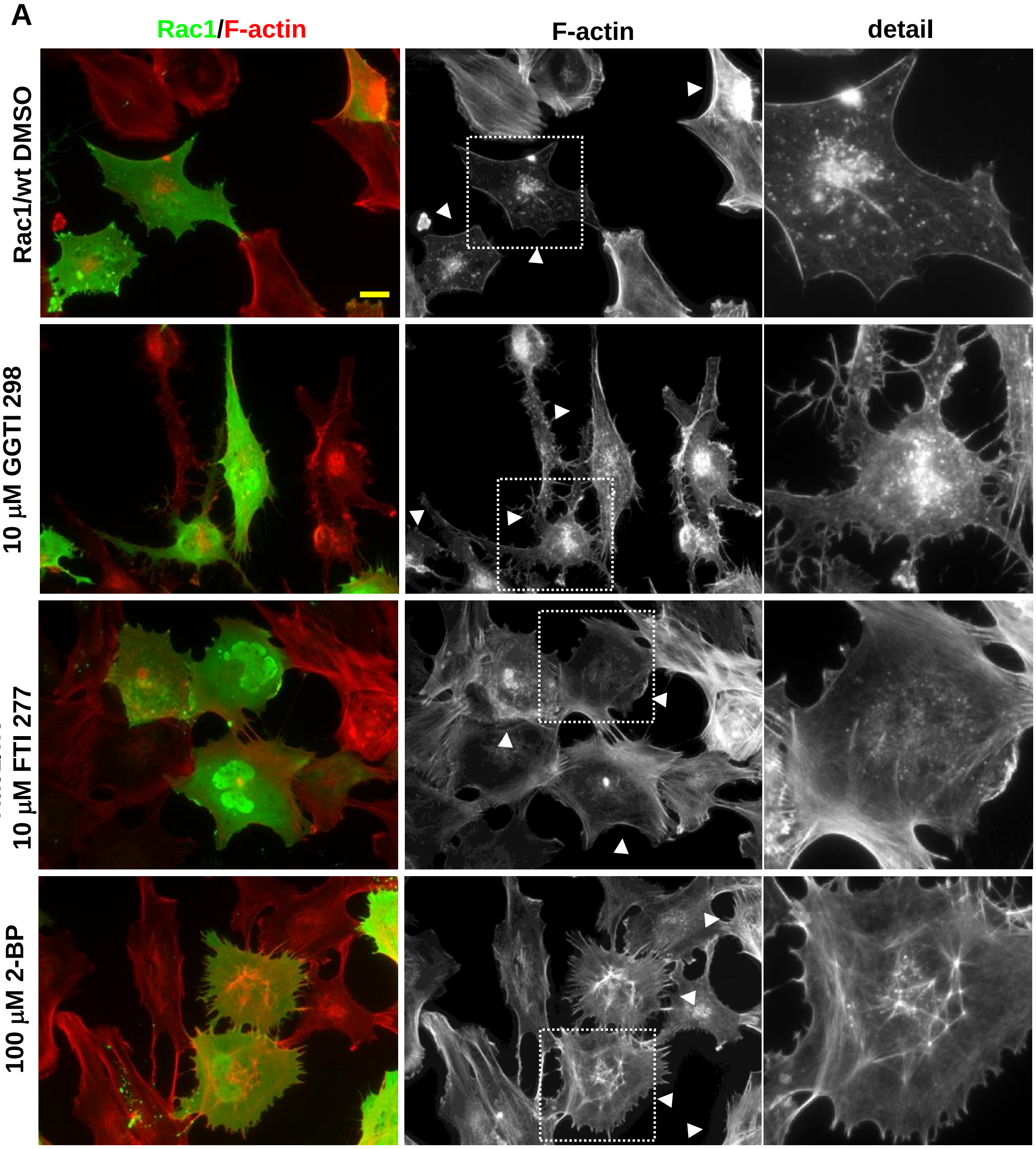

Supplementary Figure 11

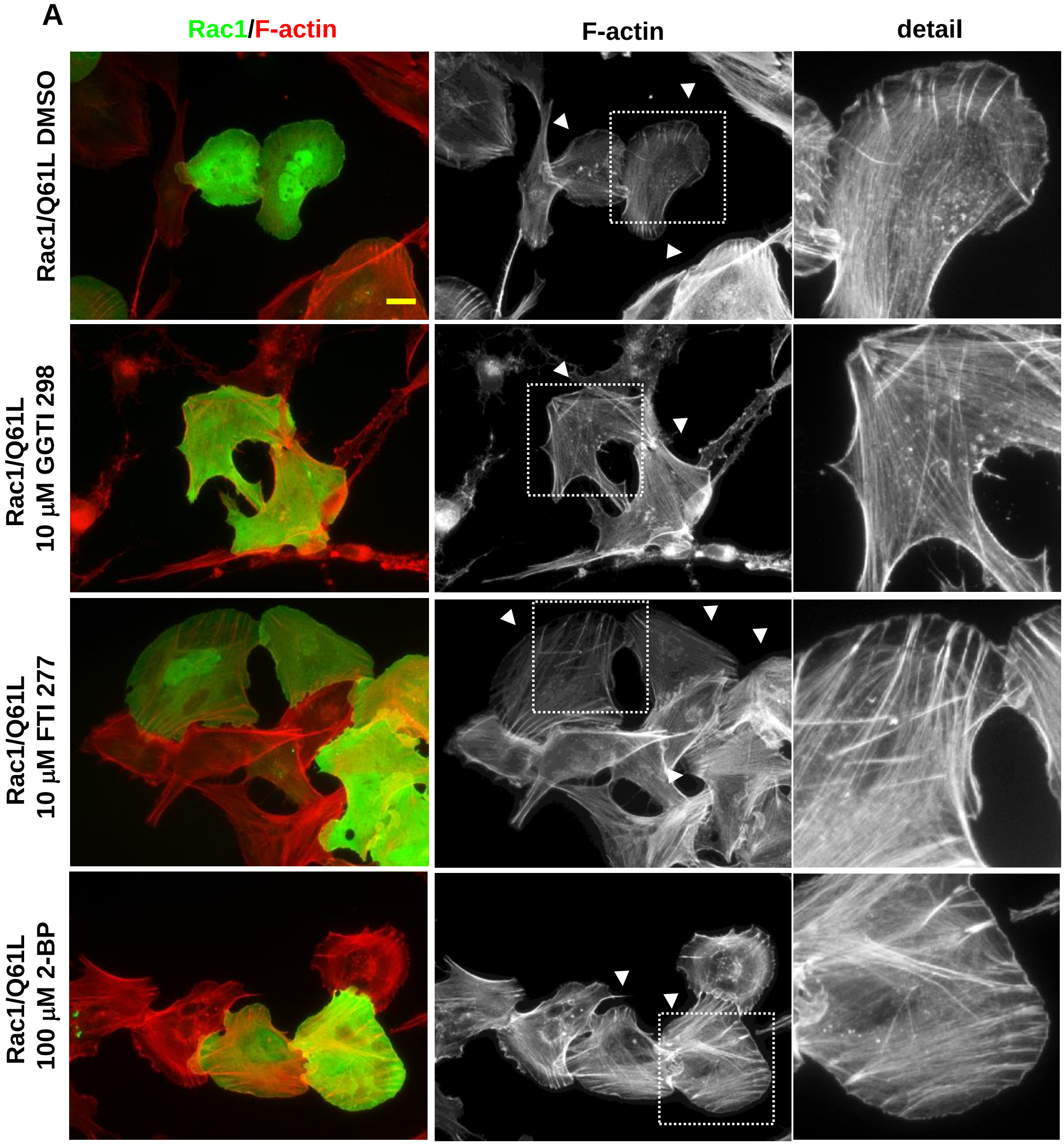

Supplementary Figure 12

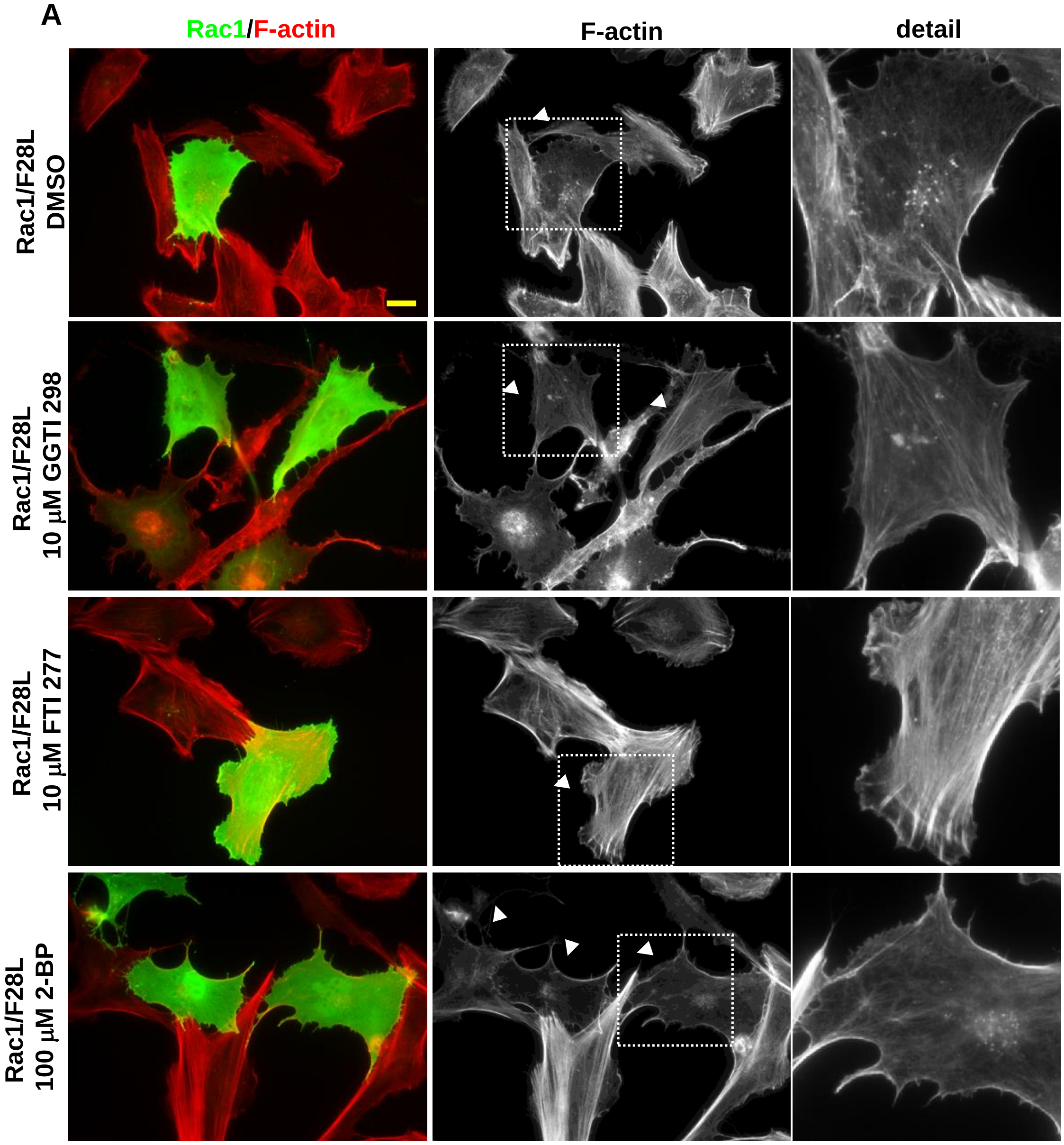

Supplementary Figure 13

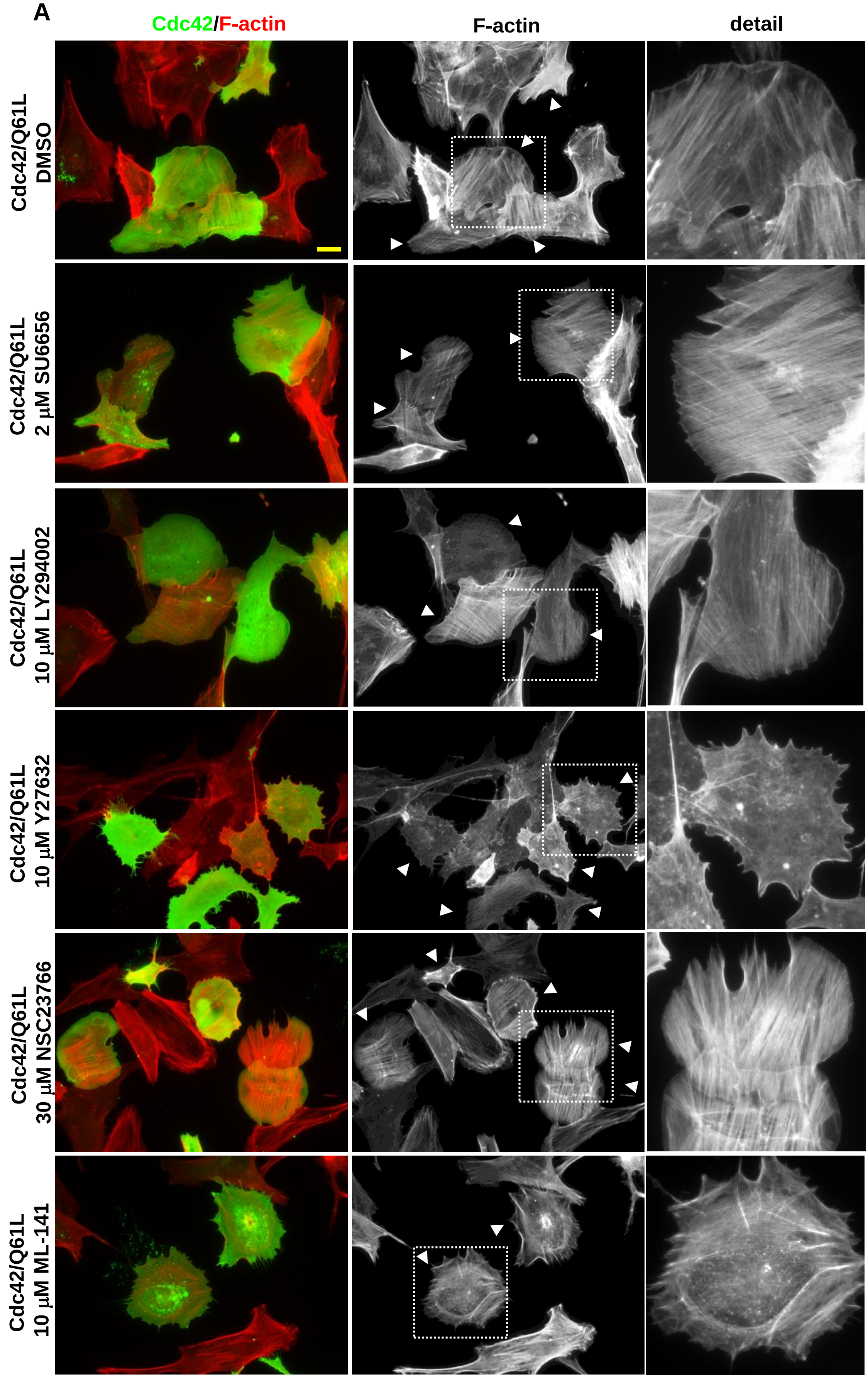

Supplementary Figure 14

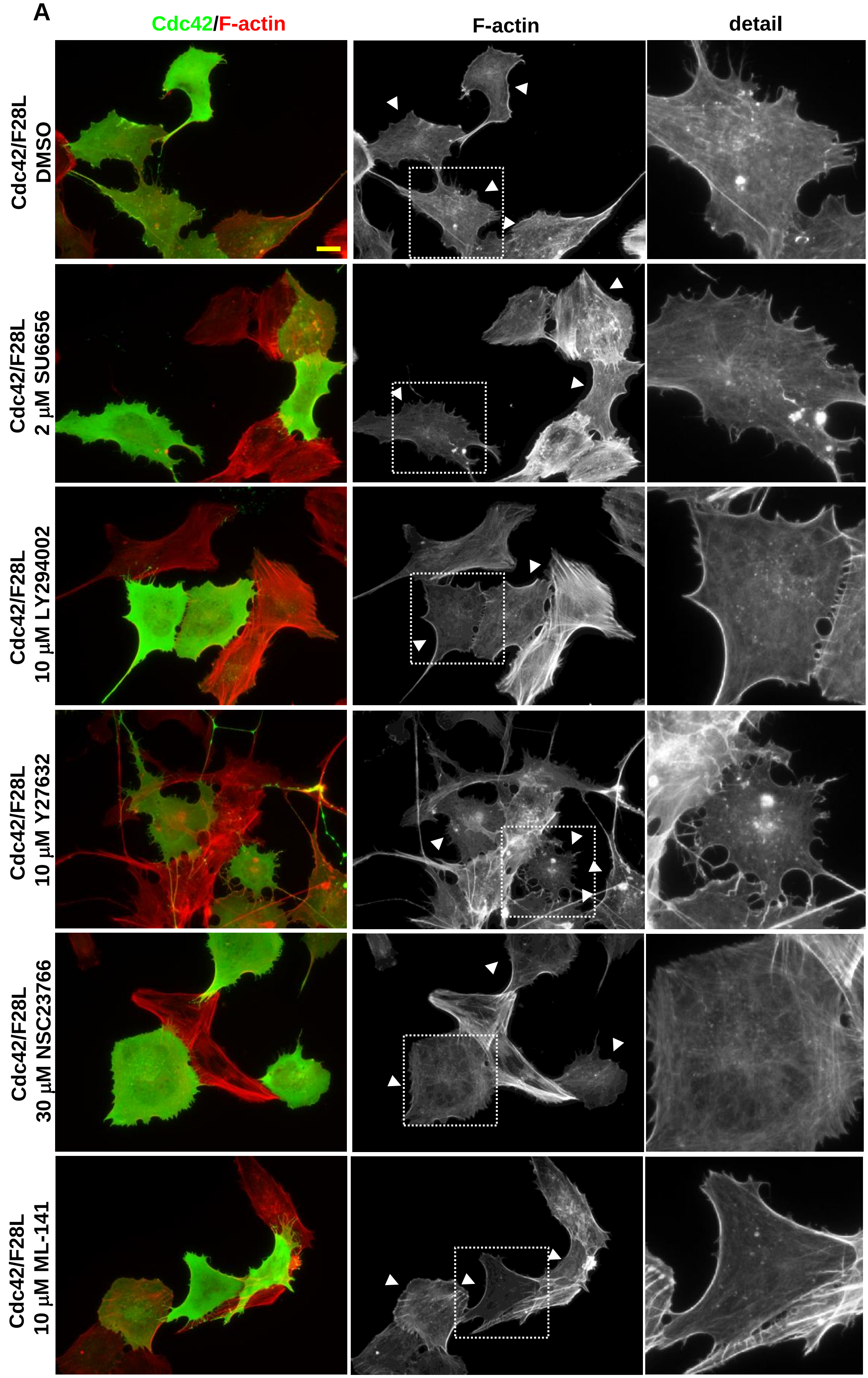

Supplementary Figure 15

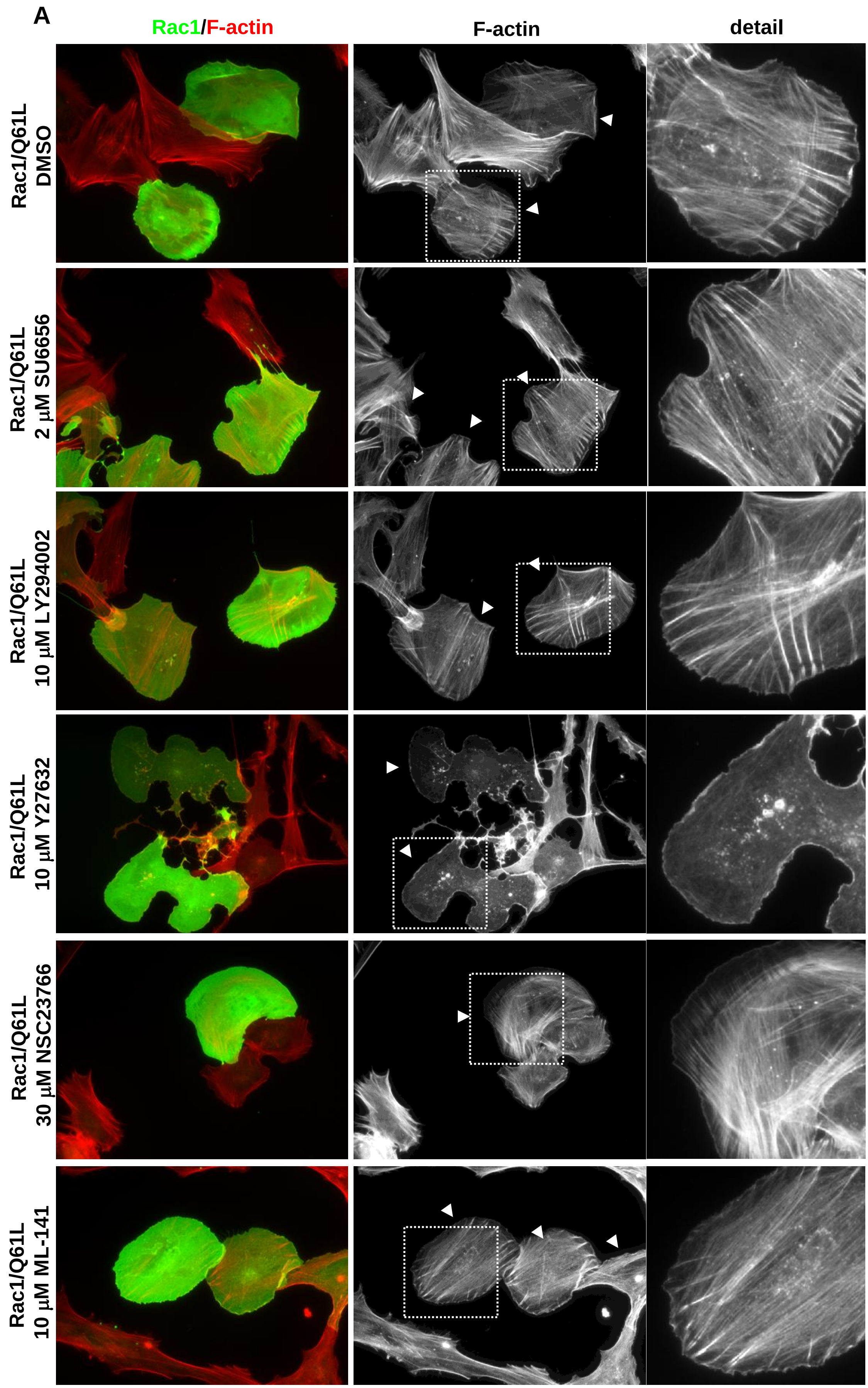

Supplementary Figure 16

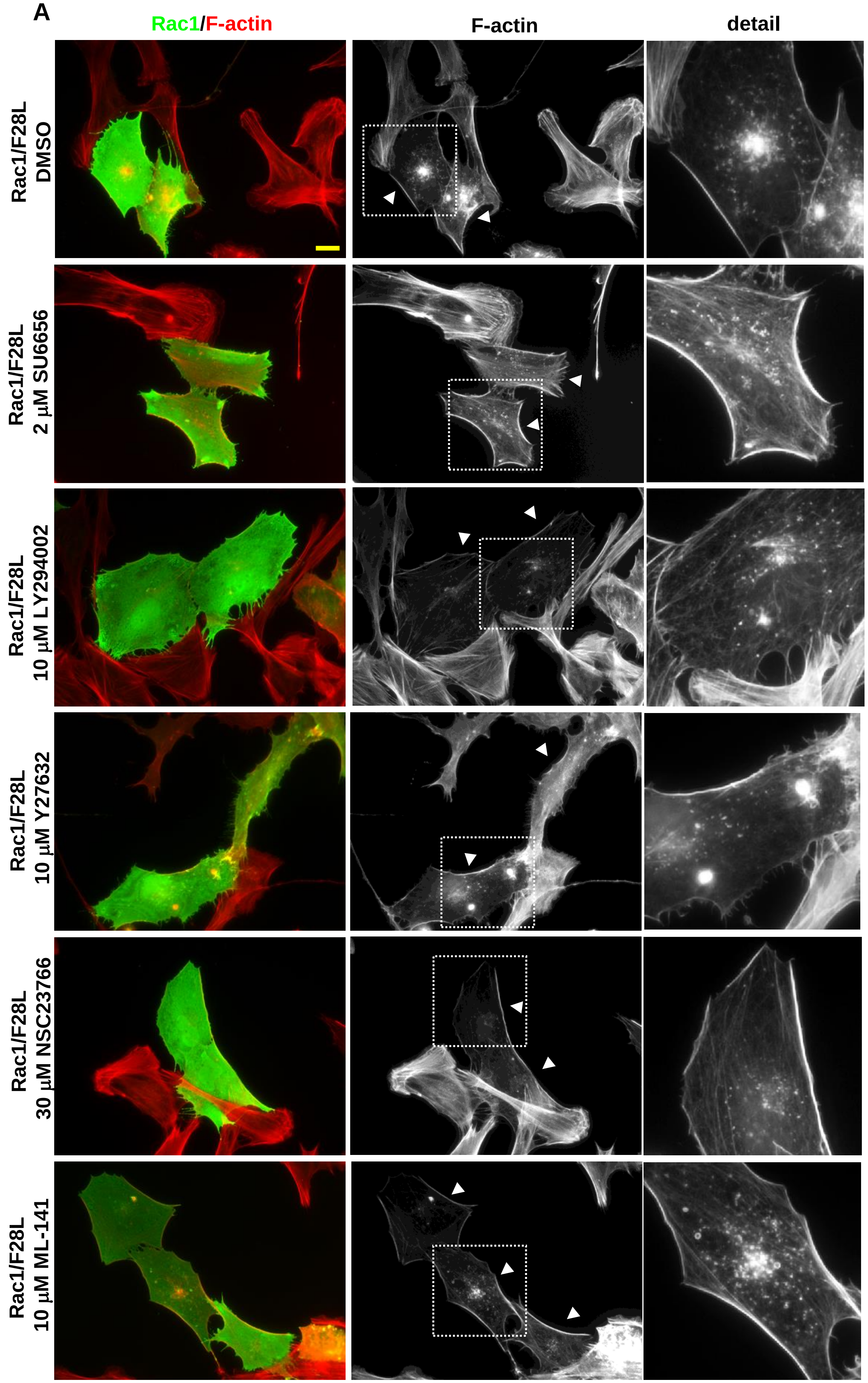

Supplementary Figure 17

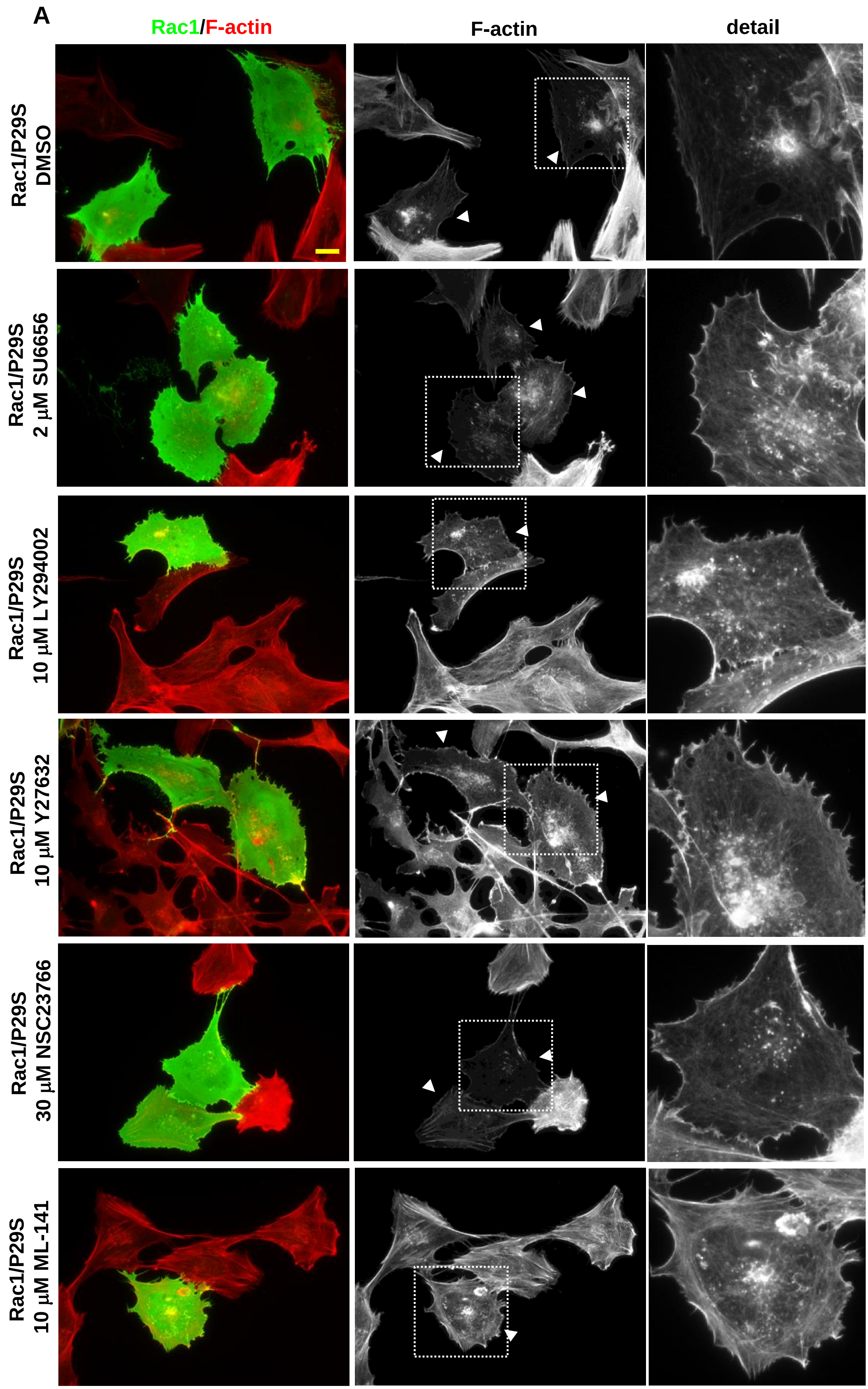

Supplementary Figure 18

A

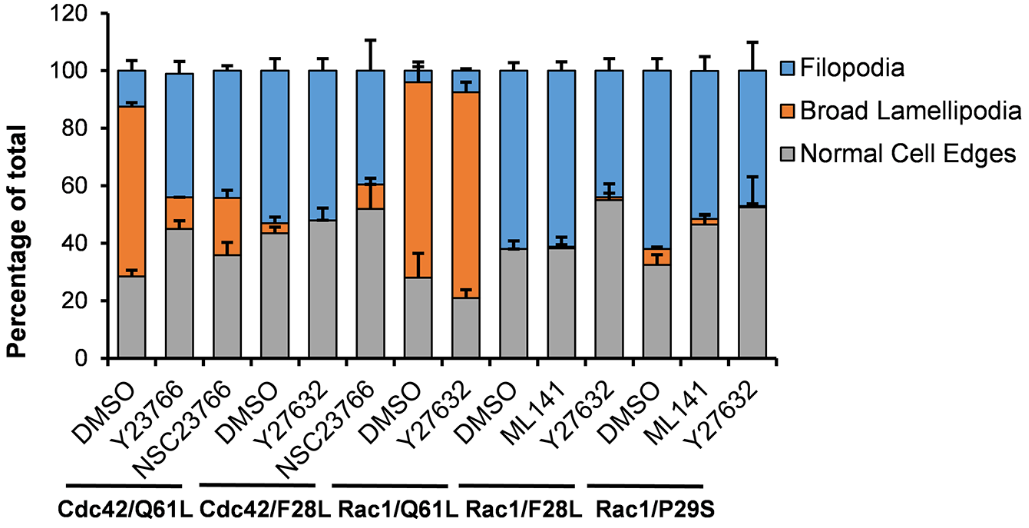

B

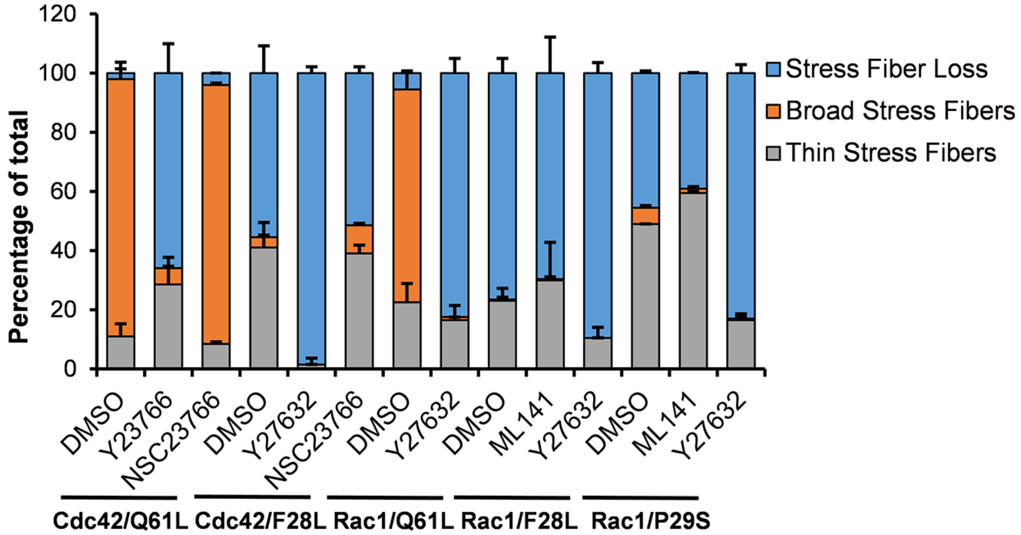

Supplement: Supplementary file 1 [file cells-08-00759-s001.zip › cells-522436-supplementary for proofreading/cells-522436-supplementary-figures.pdf]
